# Bladder cancer in cancer patients: population-based estimates from a large Swedish study

**DOI:** 10.1038/sj.bjc.6605325

**Published:** 2009-09-15

**Authors:** J Lorenzo Bermejo, J Sundquist, K Hemminki

**Affiliations:** 1 Institute of Medical Biometry and Informatics (IMBI), University Hospital Heidelberg, 69120 Heidelberg, Germany; 2 Division of Molecular Genetic Epidemiology, German Cancer Research Centre (DKFZ), 69120 Heidelberg, Germany; 3 Center for Primary Health Care Research, 20502 Malmö, Sweden

**Keywords:** urinary bladder cancer, second primary tumours, recurrence risk, population based studies

## Abstract

**Background:**

This study quantified the risk of urinary bladder neoplasms in cancer patients taking into account the age at first diagnosis, the gender of the patients and the lead time between diagnoses.

**Methods:**

We used standardised incidence ratios (SIRs) to compare the incidence of bladder tumours in 967 767 cancer patients with the incidence rate in the general Swedish population. A total of 3324 male and 1560 female patients developed bladder tumours at least 1 year after first cancer diagnosis.

**Results:**

After bladder and renal pelvis cancers, the SIRs of bladder neoplasms were higher in female than in male patients. Men affected by lung, stomach and larynx tumours belonged to the population at high risk for bladder cancer. Treatment of breast, ovarian and cervical cancers seems to contribute to the subsequent development of bladder neoplasms. Long latencies (16–25 years) were observed after testicular, cervical and endometrial cancers. Detection bias had an important role after prostate cancer. Chemotherapy with cyclophosphamide and cisplatin, and also radiotherapy, seem to increase the risk of subsequent neoplasms in the bladder.

**Conclusions:**

These population-based results may help urologists to assess the risk of bladder neoplasms in cancer survivors. Our data should guide ongoing studies that investigate the effectiveness of bladder cancer screening in cancer patients.

The investigation of the development of urinary bladder neoplasms in cancer patients may provide unique clues to advance the understanding of the aetiology of bladder cancer. Tobacco smoking, occupational exposure to aromatic and heterocyclic amines, and probably chronic bladder infections are established risk factors for bladder cancer (Nordlund *et al*, 1997; Brennan *et al*, 2000, 2001; IARC, 2004; Gandini *et al*, 2008; Kiemeney *et al*, 2008). Variants in genes coding for xenobiotic-transforming enzymes and polymorphisms in DNA repair genes may also modify cancer susceptibility (Easton *et al*, 2007; Kellen *et al*, 2007; Murta-Nascimento *et al*, 2007b; Sanderson *et al*, 2007; Andrew *et al*, 2008). In addition to detection bias and risk factors shared by cancer of distinct types, for example, tobacco smoking is a risk factor for both lung and bladder cancers, second primary neoplasms may be related to the treatment of the first tumour. Radiotherapy to the pelvic area and chemotherapy with cyclophosphamide increase the risk of bladder cancer (Murta-Nascimento *et al*, 2007a).

After laryngeal cancer, bladder cancer shows the highest excess of risk for men compared with that for women in Sweden and the excess risk increases with age (National Board of Health and Welfare, 2007; Bermejo *et al*, 2008). In Sweden in 2005, the incidence ratio of bladder cancer in men compared with that in women was 3.1 in the age band 50 to 54 years and this ratio increased to 4.1 for the age band 80–84 years (Engholm *et al*, 2007). Recent meta-analyses imply that, at least in the western world, smoking can only partially explain this gender difference in the incidence of bladder cancer (Hemelt *et al*, 2008).

The objective of this article was to examine the risk of bladder neoplasms after cancer diagnosis, taking into account the gender of the patients, the age of diagnosis of the first cancer and the time since first diagnosis. These data may be relevant for clinical counselling, future development of screening programmes, and to advance the understanding of the aetiology of the disease. The present results were based on around 3000 male and 1500 female cancer patients who developed bladder tumours at least 1 year after first cancer diagnosis. In addition to the large sample size from a single country, the nationwide complete coverage and the reliability of cancer data represented important advantages of this study.

## Material and methods

The Swedish Family-Cancer Database includes persons born in Sweden after 1931, totalling more than 11.8 million persons and more than 1.2 million tumour notifications – for a detailed description of the Database and its last update see reference Bermejo *et al* (2008). Cancer cases were retrieved from the Swedish Cancer Registry, which relies on separate compulsory notifications of cases from clinicians who diagnosed a neoplasm and from pathologists/cytologists. Second cancers were classified as such by the Cancer Registry, including synchronous tumours. The percentage of histologically or cytologically verified cases of cancer has been close to 100% (National Board of Health and Welfare, 2007). Unfortunately, the Swedish Cancer Registry lacks historic clinical and treatment data. In this study, 967 767 cancer patients were followed up from first cancer diagnosis until death, recurrence, detection of a second primary cancer, emigration or 31 December 2006, whichever came first.

The incidences of second primary urinary bladder malignancies among cancer patients were compared with the rates of first primary bladder cancers in the general Swedish population by standardised incidence ratios (SIRs) and 95% confidence intervals (CIs), adjusting for covariates age (5-year bands), sex, socioeconomic index (six groups), region (four groups) and calendar year (1961 to 1964, 1965 to 1969, and so on to 2000 to 2006). Separate analyses were carried out according to age at first cancer diagnosis (before age 20 years, 20 to 39, 40 to 59 and after 60 years). The SIR applies indirect standardisation, which is particularly suitable for cells with small numbers of subjects. In this method the observed number of cases is divided by the expected number of cases, calculated from the whole background population of 11.8 million individuals. The investigation of 36 types of cancer may result in false positive associations due to multiple comparisons. To alleviate this problem, associations were reported according to 0.05 and 0.01 significance levels. Kidney cancers were separated into cancers of the renal pelvis (International Classification of Diseases, 7th revision (ICD7)=1801) and the renal parenchyma (ICD7=1800). Cancer types were classified as ‘recurrent sites’ (urinary bladder and renal pelvis), ‘smoking-related sites’ and ‘non-smoking-related sites’.

## Results

Table 1 shows gender-specific SIRs of bladder tumours in cancer patients. Results are presented for ‘any time’ and ‘at least 1 year’ between the two diagnoses. For example, 14 women developed bladder cancer after upper aerodigestive tract cancer. Their risk of bladder cancer was 2.22 times higher than the averaged risk in the general female population. When follow-up was started 1 year after first diagnosis, the number of patients decreased to 13, the SIR was 2.54. To limit the possible effect of surveillance bias due to first diagnosis, following description focuses on tumours diagnosed at least one year apart. Significant findings at the 0.01 confidence level are underlined in Table 1.

Among female patients, the highest increases in risk were observed after renal pelvis (SIR=61.4) and bladder (SIR=10.5) neoplasms. The SIRs higher than 3.0 were found in women affected by cancer in the cervix, other female genital organs, stomach and endometrium. Statistically significant (*P*<0.01) increases were also observed after ovarian, lung, rectal, colonic and breast cancers. The SIR after cancer at any smoking-related site was 3.84 and it equalled SIR=1.77 after any non-smoking-related site. The averaged SIR of bladder neoplasms in women 1 year after diagnosis of any type of cancer was 2.22. Among men, the highest increases in the risk of bladder cancer were also observed after renal pelvis (SIR=23.4) and bladder (SIR=5.27) neoplasms. Interestingly, SIRs of recurrent neoplasms were significantly lower for male than for female patients. The highest SIRs in men were found after liver, eye and lung cancers. The SIRs in Table 1 permit to compare the risks of bladder neoplasms after diagnosis of different types of cancer. For example, men affected by lung cancer were at a higher risk of bladder tumours than prostate cancer patients (disjoint CIs).

In addition to shared risk factors, the diagnosis of two cancers in the same individual may be related to the treatment of the first tumour. Results in Table 2, based on tumours diagnosed at least 1 year apart, may help to discriminate between these two components. For example, Table 1 shows the SIR of 2.57 after stomach cancer in men. Bladder cancer patients did not show an increased risk of stomach cancer, thus favouring the effect of stomach cancer treatment over shared risk factors. The association between colorectal and bladder cancers was noticed in both directions, with similar risk increase in women. In men, the SIRs after bladder cancer were statistically lower than the SIRs in the opposite sequence. The association between lung and bladder tumours was observed in both directions. Female patients diagnosed with bladder cancer did not show an increased risk of breast and ovarian tumours, and a preventive effect was found for cervical cancer (SIR=0.41). These data probably signalise a contribution of treatment of breast, ovarian and cervical cancers to the development of subsequent bladder neoplasms. The SIR of endometrial cancer after bladder tumours was 4.83, higher than the SIR of bladder cancer after endometrial cancer. The relative risk of prostate neoplasms in bladder cancer patients (SIR=1.41) was significantly lower than the risk increase in the opposite direction.

**Figure 1:**
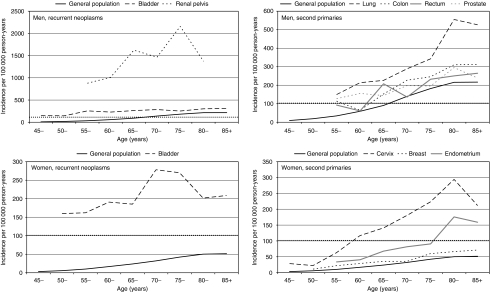
Age- and gender-specific incidence rates of urinary bladder cancer in the general population and in patients affected by selected types of cancer. Only tumours diagnosed at least 1 year apart were included in the calculations. Results are presented for cancer sites with at least 100 patients affected by bladder cancer as second malignancy and for age intervals with at least five observed cases.

Figure 1 shows absolute incidence rates for cancer types with at least 100 patients affected by second bladder neoplasms, and for age intervals with at least five observed cases. The incidence rates of bladder cancer in the general population are also shown. Among men, the highest recurrence rate was observed among renal pelvis cancer patients (around 2 cases per 1000 person-years in the age band 75 to 85 years). The incidence was higher than 200 per 10^5^ person-years in men with bladder cancer older than 55 years. Men affected by colon cancer reached an incidence of 300 per 10^5^ person-years at age 80–85 years. More modest incidences were found among female patients. Recurrence rates after urinary bladder cancer were the highest (around 270 cases per 10^5^ person-years) at age 70–79 years. Cervical cancer survivors showed the highest rate of bladder neoplasms at age 80–85 years. The estimated incidence of second bladder neoplasms in endometrial cancer patients was around 180 per 10^5^ person-years in the age period of 80–85 years.

Consideration of the age of diagnosis of the first malignancy, together with patient's age, may result in more accurate estimates of the risk to develop secondary bladder neoplasms. Table 3 shows SIRs according to these categories. Results are only presented for cancer types with at least 100 patients affected by second bladder tumours, for testicular cancer and for cells with observed cases. Only tumours diagnosed at least 1 year apart were included in the calculations.

The SIRs of recurrent bladder neoplasms in bladder cancer patients decreased with age in both men and women, and they were markedly higher in women compared with those in men. The SIRs after renal pelvis cancer also decreased with increasing ages and with increasing lead times. The SIRs in lung cancer patients showed a U-shaped pattern. Cervical cancer patients showed the highest SIRs of bladder neoplasms in the age period of 56–65 years (SIR=5.83) and women diagnosed with cervical cancer at age 40–59 years showed the highest increase in risk of bladder neoplasms 16–25 years after the first diagnosis. The SIR after renal parenchyma cancer was almost constant.

Colorectal cancer patients showed decreasing SIRs with increasing ages. The SIRs according to the age of diagnosis of the first malignancy are presented in the three right columns of Table 3. For example, the SIR of bladder cancer was 1.63 in patients aged 66–75 years who were diagnosed with colorectal cancer at the age of 40–59 years. Women affected by breast cancer showed decreasing SIRs with increasing ages. The risk pattern was bimodal in women diagnosed at age 40–59 years, with the first maximum shortly after first diagnosis and a second maximum after 16–25 years. Long latency times were also noticed in women affected by endometrial cancer. Prostate cancer patients showed decreasing SIRs with increasing ages, and with increasing time since first diagnosis. By contrast, the pattern of risk in men affected by testicular cancer favoured treatment effects. Men affected by non-Hodgkin lymphoma showed SIR maxima in the age intervals ‘before age 45 years’, ‘56–65 years’ and ‘after 85 years of age’.

## Discussion

In the near future, urologists will face an increasing number of cancer survivors visiting their practices. This study investigates the risk of second bladder neoplasms in cancer patients. We used two different strategies to discriminate between risk factors shared by subsequent malignancies and the effect of first cancer treatment. First, we compared the SIRs of bladder tumours in cancer patients with the SIRs of second cancers in patients affected by bladder tumours. The second approach investigated the pattern of risk after first tumours: asynchronous constant or decreasing relative risks may favour shared risk factors, whereas increasing risks with increasing lead time should point to treatment effects (Heard *et al*, 2005). There is an excellent literature on the development of bladder tumours after specific types of cancer, for example after prostate, oesophageal, testicular, cervical and lung tumours, and after lymphomas (Kaldor *et al*, 1987; Pettersson *et al*, 1990; Pathak *et al*, 1992; Bjorge *et al*, 1995; Bokemeyer and Schmoll, 1995; Travis *et al*, 1995, 1997; Levi *et al*, 1996, 1999; Fisher *et al*, 1997; Teppo *et al*, 2001; Dores *et al*, 2002; Brennan *et al*, 2005; Heard *et al*, 2005; Liauw *et al*, 2006; Bostrom and Soloway, 2007; Chaturvedi *et al*, 2007; Kellen *et al*, 2007; Landgren *et al*, 2007; Muller *et al*, 2007; Chuang *et al*, 2008; Singh *et al*, 2008). The main advantage of this study was the simultaneous investigation of the most common types of cancer before bladder tumours using a uniform reference population. Unfortunately, information on disease grade, treatment and smoking history was not available. The number of affected patients was small for some combinations of first cancer types and age categories, and chance due to multiple comparisons probably explains some of the detected associations.

In addition to lung and bladder cancers, tobacco smoking has been related to an increased risk of kidney, oral cavity, larynx, oesophagus, pancreas and stomach tumours (IARC, 2004). Table 4 shows a summary of risk factors that may result in the development of urinary bladder neoplasms in cancer patients. Although this list is not comprehensive and some readers would attribute a rather minor effect to some of the enumerated factors, we consider that the table may facilitate the interpretation of present results in the following discussion.

With the exception of pancreatic cancer, characterised by a very poor prognosis, our data confirmed that patients affected by tobacco-related malignancies were at an increased risk of bladder tumours. Tobacco smoking confers the highest risk to lung cancer, which was reflected in SIRs of 2.21 for women and 2.91 for men. The estimated SIRs were in agreement with a previous Finnish study that investigated the risk of new primary tumours in lung cancer patients and suggested that the increased use of cytostatic drugs may increase the risk of second tumours (Teppo *et al*, 2001). Recent studies have shown that patients with non-muscle invasive bladder cancer suffer a high incidence of mortality from lung cancer and they might constitute a suitable population for a lung screening trial (Rusthoven *et al*, 2008). These data indicate that men affected by other tobacco-related cancers, in particular by stomach, larynx and lung tumours, belong to the population at high-risk for bladder cancer.

We found a borderline increased risk of bladder cancer (SIR=1.61) in women affected by non-Hodgkin lymphoma, the SIR was 1.95 for male patients. These estimates were slightly higher than the overall SIR of 1.50 found in an international study, which included part of the Swedish cohort (Brennan *et al*, 2005; Hemminki *et al*, 2008). International studies take benefit from a large number of patients, but standard registration and homogeneity in treatment and exposure were important plus factors of this study. The increase in risk with time since diagnosis in the two studies underlines the effect of treatment for non-Hodgkin lymphoma on the risk of second bladder neoplasms. Treatment regimes for non-Hodgkin lymphoma typically involve chemotherapy with cyclophosphamide, which has been associated with a 4.5- to 10-fold increased risk of bladder cancer (Travis *et al*, 1995). Localised radiotherapy may be also applied for low-grade subtypes of lymphoma, which may additively interact with cyclophosphamide treatment. The present simultaneous consideration of age at first diagnosis and time since diagnosis suggested that treatment effects are particularly important when lymphomas are diagnosed after 40 years of age. Cyclophosphamide is also used, often in combination with other drugs, to treat leukaemia and this may contribute to the increased SIRs we found among male leukaemia patients (Simister, 1971).

Women affected by cervical cancer showed a large increase in their risk of bladder tumours, and reversed analyses suggested an important contribution of cervical cancer treatment. A recent study that included Swedish data found an increased risk in patients treated with and without radiotherapy (Chaturvedi *et al*, 2007). The SIRs around 4 have been reported in studies based on women from the United States (Fisher *et al*, 1997). These data were in agreement with these studies and indicated that survivors of cervical cancer older than 65 years clearly belong to the population at high-risk for bladder cancer. In general, cervical cancer patients smoke more than women in the general population, and some of the malignancies that were diagnosed close in time were probably related to tobacco smoking. We observed that the risk of bladder cancer increased among women affected by tumours at other genital organs than the cervix, for example, the endometrium.

Previous studies have shown that men with testicular cancer continue to be at significantly an elevated risk of second bladder cancer for more than two decades after initial diagnosis (Travis *et al*, 1997). These data corroborated this result and showed that, even 26–35 years after testicular cancer diagnosed before the age of 40 years, the SIR was around 6. Although the estimated dose of radiation to the bladder is generally higher after non-seminoma than after seminoma treatment, studies that have taken the histological type into account show similar SIRs after the two types of germ-cell tumours. The use of radiotherapy fields to treat testicular cancer has decreased in recent decades and lower doses are used now. However, it has been hypothesised that cisplatin may act as radiation enhancer and contribute to a shortened latency periods for radiogenic bladder cancer. Histological information is available in the Swedish Family-Cancer Database, and future studies may add population-based evidence to this respect.

The within-patient clustering of bladder and prostate tumours has been extensively explored (Liauw *et al*, 2006; Bostrom and Soloway, 2007; Kellen *et al*, 2007; Bostrom *et al*, 2008; Singh *et al*, 2008). In contrast with testicular cancer, which showed increasing SIRs with increasing latency times, the SIRs of bladder neoplasms decreased with the time after diagnosis of prostate cancer. The reversed analyses also suggested a major contribution of risk factors shared by bladder and prostate tumours. Relevant to the urological clinical practice, our results indicated that men older than 60 years affected by prostate cancer show an excess of second primary bladder tumours. Previous studies have shown that detection bias may have an important role in the first year of follow-up, and our results confirmed this statement (Kellen *et al*, 2007). Out of 1721 patients affected by bladder after prostate cancer, (1721−1000)/1721=42% were diagnosed with the two tumours within 1 year. A similar proportion was observed when the opposite sequence was investigated. Prostate cancer radiotherapy has been suggested to induce secondary malignancies in the bladder, but if radiation were a central issue, SIRs would increase with follow-up time, and this trend was not observed (Liauw *et al*, 2006).

Mutations in DNA repair genes (*XRCC3*) and variants in genes coding for xenobiotic-transforming enzymes (*NAT2*, *GSTM1*, *GSTP1* and *NQO1*) have been shown to modify the susceptibility to bladder cancer (Chao *et al*, 2006; Chaturvedi *et al*, 2007; Easton *et al*, 2007; Figueroa *et al*, 2007; Kellen *et al*, 2007; Sanderson *et al*, 2007; Murta-Nascimento *et al*, 2007b). These variants may be associated with an increased risk of cancer at additional sites. For example, two meta-analyses that explored the relevance of *GSTM1* null status on stomach cancer found a modest risk increase (La Torre *et al*, 2005; Saadat, 2006). A recent genome-wide association study identified a novel variant, which confers an increased risk for both urinary bladder and lung cancers (Kiemeney *et al*, 2008). These polymorphisms are relatively common in Swedes, but the low penetrances conferred by the risk alleles (genotype relative risks between 1.2 and 1.5) result in a limited contribution of the variants to the increased risk of bladder neoplasms among cancer patients.

These data may be used to design future studies that investigate the effectiveness of bladder cancer screening in cancer patients. Although the definition of the population at high risk depends on multiple interacting factors, men older than 60 years of age and a smoking history have been traditionally considered to be at high risk (Grossman *et al*, 2005). The incidence of bladder cancer among men of 60 years of age in the Swedish Family-Cancer Database was around 60 cases per 10^5^ person-years. The combination of this figure with a smoking prevalence of 40% (Stegmayr *et al*, 2005), and with an odds ratio of bladder cancer for smokers versus non-smokers of five, results in an incidence of bladder cancer of 115 cases per 10^5^ person-years for men older than 60 years with a smoking history. We drew a horizontal line on Figure 1 to characterise the groups of high-risk patients according to this criterion. As expected, bladder and renal pelvis cancer patients show very high recurrence risks. After lung cancer, for every 1000 survivors, approximately 5 developed secondary bladder cancer in the age interval 80–85 years. The threshold of 100 cases per 10^5^ person-years was reached at the age of 60–65 years by male patients with colorectal and prostate cancers. Among women, high-risk groups consisted of cervical cancer patients older than 55 years and endometrial cancer patients older than 75 years. Note that absolute incidences among cancer patients can be approximated by combining the SIRs in Table 3 with population-specific incidence rates.

The present data may help urologists to assess the risk of bladder neoplasms in cancer survivors, who are going to ask for clinical advice with increasing frequency in the near future. Our results should also guide future studies that investigate the effectiveness of bladder cancer screening in cancer patients. The contribution of known genetic variants to the observed associations is probably minor. Treatment of the first malignancy, in particular chemotherapy with cyclophosphamide and cisplatin, and also radiotherapy, probably have a larger impact. Smoking can be instrumental in the development of bladder cancer after tumours in tobacco-related sites.

## Figures and Tables

**Table 1:** Number and SIRs of second bladder tumours in cancer patients

	**Women**				**Men**			
	**Time between two diagnoses**				**Time between two diagnoses**			
	**Any time**		**At least 1 year**		**Any time**		**At least 1 year**	
**First cancer type**	***N***	**SIR (95% CI)**	***N***	**SIR (95% CI)**	***N***	**SIR (95% CI)**	***N***	**SIR (95% CI)**
*Recurrent neoplasms*								
Urinary bladder	189	**19.6** (17.0–22.7)	132	**10.5** (8.87–12.5)	655	**6.47** (5.98–6.99)	501	**5.66** (5.19–6.19)
Renal pelvis	93	**205** (167–251)	48	**61.4** (46.3–81.6)	187	**61.8** (53.5–71.3)	108	**23.4** (19.4–28.3)
								
*Smoking-related cancer sites (excluding recurrent neoplasms)*								
Upper aerodigestive tract	14	**2.22** (1.32–3.76)	13	**2.54** (1.47–4.37)	105	1.18 (0.98–1.43)	93	1.18 (0.96–1.45)
Oesophagus					10	**2.17** (1.17–4.03)	6	**2.64** (1.19–5.87)
Stomach	19	**2.87** (1.83–4.51)	16	**3.29** (2.01–5.37)	103	**2.56** (2.11–3.11)	80	**2.57** (2.06–3.20)
Anus	7	**3.16** (1.51–6.63)	5	2.12 (0.88–5.09)	7	**2.75** (1.31–5.77)	5	**2.63** (1.10–6.33)
Pancreas	1	0.69 (0.10–4.93)			9	1.47 (0.77–2.83)	4	0.67 (0.25–1.80)
Nose	2	1.23 (0.31–4.91)	1	0.51 (0.07–3.60)	13	1.60 (0.93–2.75)	11	1.48 (0.82–2.66)
Larynx					96	**2.32** (1.90–2.84)	88	**2.34** (1.90–2.89)
Lung	24	**2.69** (1.80–4.02)	18	**2.21** (1.39–3.51)	151	**2.95** (2.51–3.46)	101	**2.91** (2.40–3.54)
Cervix	278	**5.60** (4.97–6.31)	258	**5.45** (4.82–6.17)				
Renal parenchyma	38	**2.82** (2.05–3.88)	25	**1.57** (1.06–2.33)	119	**2.33** (1.94–2.78)	84	**1.38** (1.12–1.71)
Any smoking-related	383	**4.01** (3.62–4.43)	336	**3.84** (3.45–4.27)	613	**2.08** (1.92–2.25)	472	**1.75** (1.60–1.91)
								
*Non-smoking-related cancer sites*								
Salivary glands	5	1.85 (0.77–4.45)	5	1.97 (0.82–4.75)	18	**1.59** (1.00–2.53)	15	1.48 (0.89–2.46)
Small intestine	4	1.06 (0.40–2.83)	4	1.22 (0.46–3.26)	16	1.43 (0.88–2.34)	11	0.96 (0.53–1.72)
Colon	102	**1.87** (1.54–2.27)	89	**1.71** (1.39–2.10)	294	**1.71** (1.52–1.92)	253	**1.86** (1.65–2.11)
Rectum	43	**1.77** (1.31–2.38)	38	**1.77** (1.28–2.43)	174	**2.50** (2.15–2.90)	135	**2.63** (2.22–3.11)
Liver	6	**2.66** (1.19–5.91)	1	0.29 (0.04–2.04)	20	**14.1** (9.11–21.9)	11	**3.56** (1.97–6.43)
Breast	378	**1.77** (1.60–1.96)	352	**1.42** (1.28–1.58)	4	0.44 (0.17–1.18)	3	0.37 (0.12–1.14)
Endometrium	212	**2.80** (2.45–3.21)	206	**3.13** (2.73–3.60)				
Ovary	85	**2.63** (2.13–3.26)	82	**2.85** (2.29–3.54)				
Other female genital	25	**4.45** (3.01–6.59)	19	**3.99** (2.54–6.25)				
Prostate					1721	**3.99** (3.81–4.19)	1000	**2.05** (1.93–2.19)
Testis					70	**2.91** (2.30–3.68)	65	**2.65** (2.08–3.38)
Other male genital					24	**1.90** (1.28–2.84)	17	1.39 (0.86–2.24)
Melanoma	39	1.24 (0.90–1.70)	37	1.26 (0.92–1.75)	139	**1.29** (1.10–1.53)	127	**1.36** (1.14–1.62)
Skin, squamous cell	52	**1.66** (1.26–2.18)	46	**1.35** (1.01–1.80)	258	**1.17** (1.03–1.32)	226	**1.27** (1.12–1.45)
Eye	2	0.63 (0.16–2.53)	2	0.66 (0.16–2.64)	16	**6.00** (3.68–9.80)	13	**3.09** (1.80–5.33)
Nervous system	33	**1.44** (1.02–2.02)	31	**1.42** (1.00–2.01)	59	1.13 (0.87–1.45)	52	1.08 (0.82–1.41)
Thyroid gland	15	0.93 (0.56–1.55)	15	0.97 (0.59–1.61)	15	**2.00** (1.20–3.31)	14	**1.96** (1.16–3.31)
Endocrine glands	44	1.12 (0.84–1.51)	40	1.08 (0.79–1.47)	53	1.21 (0.93–1.59)	49	1.28 (0.96–1.69)
Bone	1	0.57 (0.08–4.07)	1	0.60 (0.09–4.29)	7	1.56 (0.74–3.27)	6	1.45 (0.65–3.23)
Connective tissue	8	1.15 (0.58–2.30)	8	1.24 (0.62–2.49)	25	**1.57** (1.06–2.32)	21	**1.53** (1.00–2.34)
Hodgkin disease	3	1.25 (0.40–3.87)	2	0.93 (0.23–3.70)	17	1.39 (0.86–2.23)	17	1.55 (0.96–2.49)
Non-Hodgkin lymphoma	29	**1.51** (1.05–2.18)	27	**1.61** (1.10–2.35)	122	**1.98** (1.66–2.37)	102	**1.95** (1.60–2.36)
Myeloma	8	1.08 (0.54–2.17)	4	0.69 (0.26–1.84)	18	0.60 (0.38–0.95)	14	0.47 (0.28–0.79)
Leukaemia	22	**2.18** (1.44–3.32)	17	1.39 (0.87–2.24)	92	**1.83** (1.50–2.25)	67	**1.66** (1.31–2.11)
Any non-smoking related	1116	**1.80** (1.70–1.91)	1026	**1.77** (1.66–1.88)	3162	**2.04** (1.97–2.12)	2218	**1.52** (1.46–1.58)
								
Any site	1807	**2.47** (2.35–2.60)	1560	**2.22** (2.11–2.34)	4657	**2.40** (2.33–2.48)	3324	**1.86** (1.79–1.92)

Abbreviations: CI=confidence interval; SIR=standardised incidence ratio.

Bold type represents a significant increase at the 5% confidence level, underlined SIRs were higher than 1.00 at the 1% confidence level.

**Table 2:** Number and SIRs of second tumours in urinary bladder cancer patients

	**Women**		**Men**	
**Second cancer type**	***N***	**SIR (95% CI)**	***N***	**SIR (95% CI)**
*Recurrent neoplasms*				
Urinary bladder	132	**10.5** (8.87–12.5)	501	**5.66** (5.19–6.19)
Renal pelvis	48	**40.1** (30.0–53.5)	154	**34.5** (29.2–40.7)
				
*Smoking-related cancer sites (excluding recurrent neoplasms)*				
Upper aerodigestive tract	6	0.73 (0.33–1.63)	70	**1.28** (1.01–1.61)
Oesophagus	7	**4.53** (2.16–9.51)	47	0.87 (0.66–1.16)
Stomach	19	0.95 (0.60–1.49)	188	1.04 (0.90–1.20)
Anus	2	1.36 (0.34–5.44)	6	1.23 (0.55–2.76)
Pancreas	42	**1.54** (1.14–2.09)	120	**1.23** (1.02–1.47)
Nose	1	0.38 (0.05–2.73)	7	1.13 (0.54–2.38)
Larynx	1	0.83 (0.12–5.89)	48	**1.35** (1.02–1.79)
Lung	115	**3.32** (2.76–3.99)	645	**2.00** (1.85–2.17)
Cervix	13	0.41 (0.24–0.71)		
Renal parenchyma	16	0.60 (0.37–0.98)	91	**1.38** (1.12–1.69)
Any smoking-related	222	**1.58** (1.38–1.80)	1222	**1.50** (1.42–1.59)
				
*Non-smoking-related cancer sites*				
Salivary glands	3	2.16 (0.70–6.71)	7	1.35 (0.64–2.85)
Small intestine	5	0.82 (0.34–1.97)	32	**1.81** (1.27–2.56)
Colon	95	**1.76** (1.44–2.15)	352	**1.21** (1.09–1.34)
Rectum	46	**1.67** (1.25–2.24)	187	**1.22** (1.06–1.41)
Liver	34	**2.06** (1.47–2.88)	112	**1.96** (1.63–2.36)
Breast	195	0.98 (0.85–1.13)	5	0.95 (0.39–2.29)
Endometrium	52	**4.83** (3.68–6.34)		
Ovary	31	0.71 (0.50–1.01)		
Other female genital	6	2.04 (0.91–4.54)		
Prostate			1454	**1.41** (1.34–1.49)
Testis			2	0.98 (0.24–3.91)
Other male genital			11	0.81 (0.45–1.47)
Melanoma	23	**2.61** (1.74–3.93)	107	1.18 (0.98–1.43)
Skin, squamous cell	53	**1.64** (1.26–2.15)	272	**1.30** (1.15–1.47)
Eye	5	1.98 (0.82–4.76)	13	**3.62** (2.10–6.25)
Nervous system	24	1.24 (0.83–1.85)	70	**26.3** (20.8–33.3)
Thyroid gland	6	0.72 (0.32–1.60)	19	**1.69** (1.07–2.65)
Endocrine glands	27	**1.87** (1.28–2.72)	34	**1.72** (1.23–2.41)
Bone			3	0.40 (0.13–1.25)
Connective tissue	8	**2.18** (1.09–4.37)	27	1.30 (0.89–1.90)
Hodgkin disease	2	2.54 (0.63–10.2)	9	**6.92** (3.60–13.3)
Non-Hodgkin lymphoma	35	1.36 (0.98–1.90)	110	0.97 (0.80–1.17)
Myeloma	6	0.41 (0.18–0.92)	58	**9.97** (7.70–12.9)
Leukaemia	36	**4.53** (3.27–6.28)	121	1.16 (0.97–1.38)
Any non-smoking related	653	**1.38** (1.28–1.49)	3005	**2.85** (2.75–2.95)
				
Any site	1138	1.91 (1.80–2.03)	5060	**2.77** (2.69–2.84)

Abbreviations: CI=confidence interval; SIR=standardised incidence ratio.

Only tumours diagnosed at least one year apart were included in the calculations.

Bold type represents a significant increase at the 5% confidence level.

**Table 3:** Number and SIRs of urinary bladder tumours in cancer patients according to age and age-of-diagnosis of first malignancy

	**Age of diagnosis of the first cancer**								
		**Any**		**20–39 years**		**40–59 years**		**60+ years**	
**Cancer type (Gender)**	**Age (years)**	***N***	**SIR (95% CI)**	***N***	**SIR (95% CI)**	***N***	**SIR (95% CI)**	***N***	**SIR (95% CI)**
*Recurrent neoplasms*									
Urinary bladder (females)	46–55	6	**31.7** (14.2–70.8)	3	**74.8** (24.1–232)	3	**20.4** (6.56–63.3)		
	56–65	21	**16.4** (10.7–25.2)	4	**44.0** (16.5–118)	15	**18.0** (10.8–29.9)	2	**7.03** (1.76–28.1)
	66–75	46	**7.64** (5.71–10.2)			16	**8.53** (5.22–13.9)	30	**7.16** (5.00–10.3)
	76–85	45	**5.34** (3.97–7.16)			8	**5.76** (2.88–11.5)	37	**4.80** (3.47–6.64)
	85+	14	**3.73** (2.20–6.32)					14	**3.95** (2.33–6.70)
									
Urinary bladder (males)	<45	7	**68.6** (32.6–144)	4	**40.9** (15.3–109)	2	**99.4** (24.8– 399)		
	46–55	22	**11.2** (7.37–17.0)	8	**13.3** (6.66–26.7)	14	**11.7** (6.92–19.8)		
	56–65	94	**5.26** (4.29–6.44)	3	**6.14** (1.98–19.0)	80	**5.14** (4.12–6.40)	11	**4.40** (2.43–7.95)
	66–75	178	**2.41** (2.08–2.80)	2	**6.87** (1.72–27.5)	60	**3.07** (2.38–3.96)	116	**2.09** (1.74–2.51)
	76–85	155	**1.28** (1.09–1.50)			25	**3.62** (2.44–5.36)	130	1.16 (0.98–1.38)
	85+	45	**1.38** (1.03–1.86)			3	2.43 (0.78–7.55)	42	1.33 (0.98–1.81)
									
Renal pelvis (females and males)	<45	1	**189** (26.6–1344)			1	**618** (86.6–4413)		
	46–55	11	**114** (63.3–207)	1	**11.6** (1.63–82.4)	10	**343** (184–638)		
	56–65	27	**35.1** (24.1–51.2)	1	**15.6** (2.19–110)	13	**23.7** (13.8–40.8)	13	**164** (95.4–283)
	66–75	56	**23.3** (17.9–30.2)			5	**5.65** (2.35–13.6)	51	**32.7** (24.8–43.0)
	76–85	54	**15.6** (11.9–20.4)			3	**10.1** (3.25–31.2)	51	**17.6** (13.3–23.1)
	85+	7	**8.97** (4.27–18.8)					7	**9.34** (4.45–19.6)
									
*Smoking*-*related cancer sites (excluding recurrent neoplasms)*									
Lung (females and males)	46–55	2	**6.58** (1.65–26.3)			2	**9.90** (2.47–39.6)		
	56–65	21	**2.93** (1.91–4.50)	1	**14.8** (2.08–105)	17	**2.83** (1.76–4.55)	3	1.80 (0.58–5.57)
	66–75	46	**2.30** (1.72–3.07)			9	1.46 (0.76–2.81)	37	**2.49** (1.81–3.44)
	76–85	44	**2.21** (1.65–2.98)			5	1.92 (0.80–4.62)	39	**2.18** (1.59–2.99)
	85+	6	**2.58** (1.16–5.74)					6	**2.78** (1.25–6.20)
									
Cervix (females)	<45	1	5.62 (0.79–40.0)	1	5.63 (0.79–40.1)				
	46–55	17	**5.42** (3.36–8.76)	10	**5.70** (3.05–10.6)	7	**5.46** (2.59–11.5)		
	56–65	63	**5.83** (4.54–7.49)	23	**6.97** (4.62–10.5)	39	**4.88** (3.56– 6.7)	1	2.73 (0.38–19.4)
	66–75	89	**5.40** (4.37–6.67)	19	**6.07** (3.87–9.54)	61	**6.09** (4.73–7.85)	9	**2.59** (1.35–4.98)
	76–85	75	**5.64** (4.48–7.09)	10	**6.53** (3.51–12.2)	47	**6.72** (5.04–8.97)	18	**6.54** (4.11–10.4)
	85+	13	**4.00** (2.32–6.92)	1	**138** (19.4–981)	6	**5.33** (2.39–11.9)	6	**3.52** (1.58–7.87)
									
Renal parenchyma (females and males)	46–55	1	0.63 (0.09–4.44)			1	0.87 (0.12–6.19)		
	56–65	16	**1.65** (1.01–2.69)			13	**1.75** (1.01–3.01)	3	2.40 (0.77–7.45)
	66–75	45	**1.72** (1.28–2.30)			19	**1.99** (1.27–3.12)	26	**1.65** (1.12–2.42)
	76–85	42	**1.42** (1.05–1.93)	1	**12.8** (1.80–90.9)	6	1.13 (0.51–2.51)	35	**1.46** (1.05–2.04)
	85+	5	0.94 (0.39–2.27)			1	1.56 (0.22–11.1)	4	0.88 (0.33–2.35)
									
*Non-smoking-related cancer sites*									
Colorectum (females and males)	<45	3	**4.57** (1.47–14.2)	1	4.45 (0.63–31.6)	2	**27.1** (6.75–108)		
	46–55	10	**2.92** (1.57–5.44)	1	1.38 (0.19–9.80)	9	**4.12** (2.14–7.93)		
	56–65	35	**1.57** (1.12–2.18)	3	2.15 (0.69–6.68)	24	1.38 (0.92–2.06)	8	1.54 (0.77–3.07)
	66–75	161	**1.50** (1.28–1.75)			41	**1.63** (1.20–2.21)	120	**1.60** (1.34–1.92)
	76–85	236	**1.33** (1.17–1.52)			26	**1.60** (1.09–2.35)	210	**1.32** (1.15–1.51)
	85+	78	1.23 (0.98–1.54)			2	0.67 (0.17–2.67)	76	**1.25** (1.00–1.57)
									
Breast (females)	46–55	16	**2.10** (1.28–3.45)	2	1.29 (0.32–5.16)	14	**2.07** (1.22–3.50)		
	56–65	72	**1.71** (1.36–2.17)	3	1.01 (0.33–3.15)	59	**1.63** (1.26–2.11)	10	**2.44** (1.31–4.54)
	66–75	101	**1.25** (1.03–1.53)	2	1.10 (0.28–4.41)	38	0.89 (0.64–1.22)	61	**1.42** (1.10–1.83)
	76–85	122	**1.38** (1.15–1.66)			30	**1.75** (1.22–2.51)	92	**1.34** (1.09–1.65)
	85+	41	1.33 (0.97–1.82)			3	0.76 (0.25–2.37)	38	1.37 (0.99–1.90)
									
Endometrium (females)	<45	1	**17.7** (2.49–126)			1	**32.4** (4.54–231)		
	46–55	2	1.72 (0.43–6.87)			2	1.85 (0.46–7.42)		
	56–65	27	**2.70** (1.84–3.94)	2	**8.03** (2.01–32.1)	20	**2.35** (1.51–3.65)	5	**3.59** (1.49–8.65)
	66–75	70	**2.97** (2.34–3.76)			49	**3.58** (2.70–4.75)	21	**2.13** (1.39–3.27)
	76–85	82	**2.44** (1.96–3.04)			43	**4.19** (3.10–5.66)	39	**1.74** (1.27–2.39)
	85+	24	**2.75** (1.83–4.13)			13	**6.88** (3.98–11.9)	11	1.56 (0.86–2.84)
									
Prostate (males)	46–55	4	**6.07** (2.28–16.2)			4	**6.07** (2.28–16.2)		
	56–65	69	**3.10** (2.44–3.93)			45	**2.80** (2.09–3.75)	24	**3.21** (2.15–4.79)
	66–75	290	**1.30** (1.16–1.46)			17	1.16 (0.72–1.86)	273	**1.31** (1.16–1.48)
	76–85	495	**1.12** (1.02–1.22)			4	0.93 (0.35–2.49)	491	**1.12** (1.02–1.23)
	85+	142	1.03 (0.87–1.22)					142	1.03 (0.87–1.22)
									
Testis (males)	<45	3	2.81 (0.91–8.74)	3	**3.15** (1.01–9.78)				
	46–55	10	**2.43** (1.31–4.52)	8	**3.12** (1.56–6.24)	2	1.66 (0.42–6.65)		
	56–65	21	**2.93** (1.91–4.50)	13	**6.20** (3.60–10.7)	7	1.84 (0.88–3.86)	1	**13.1** (1.84–92.9)
	66–75	22	**2.41** (1.59–3.67)	13	**6.33** (3.67–10.9)	9	**1.96** (1.02–3.77)		
	76–85	8	**2.68** (1.34–5.37)			6	**3.72** (1.67–8.28)	2	3.23 (0.81–12.9)
	85+	1	2.99 (0.42–21.3)			1	4.07 (0.57–28.9)		
									
Melanoma (females and males)	<45	1	0.75 (0.10–5.30)	1	0.95 (0.13–6.73)				
	46–55	12	**2.91** (1.65–5.13)	3	2.24 (0.72–6.96)	9	**3.00** (1.56–5.77)		
	56–65	31	**1.45** (1.02–2.06)	4	1.13 (0.43–3.02)	25	**1.66** (1.12–2.45)	2	0.70 (0.18–2.80)
	66–75	52	1.18 (0.90–1.54)	1	0.89 (0.12–6.30)	16	**2.01** (1.23–3.28)	35	**2.03** (1.46–2.83)
	76–85	53	1.07 (0.82–1.40)			7	0.91 (0.43–1.91)	46	1.12 (0.84–1.50)
	85+	15	1.01 (0.61–1.68)			2	1.70 (0.43–6.81)	13	0.91 (0.53–1.57)
									
Skin, squamous cell (females and males)	56–65	13	1.33 (0.77–2.29)	1	0.40 (0.06–2.82)	10	1.32 (0.71–2.45)	2	1.17 (0.29–4.68)
	66–75	58	**1.44** (1.11–1.86)			13	1.15 (0.67–1.98)	45	**1.62** (1.21–2.16)
	76–85	131	**1.60** (1.34–1.90)			9	1.84 (0.96–3.54)	122	**1.59** (1.33–1.90)
	85+	70	**1.56** (1.23–1.98)			1	1.01 (0.14–7.20)	69	**1.56** (1.23–1.98)
									
Non-Hodgkin lymphoma (females and males)	<45	4	**6.43** (2.41–17.1)	3	**7.07** (2.28–22.0)				
	46–55	5	1.97 (0.82–4.75)	3	**4.83** (1.56–15.0)	2	0.96 (0.24–3.86)		
	56–65	24	**1.95** (1.31–2.91)	4	**4.36** (1.64–11.6)	19	**1.89** (1.20–2.96)	1	0.96 (0.14–6.82)
	66–75	49	**1.87** (1.41–2.48)	1	**10.8** (1.52–76.5)	19	**3.02** (1.93–4.74)	29	1.42 (0.99–2.04)
	76–85	33	1.07 (0.76–1.50)	3	**7.07** (2.28–22.0)	6	**2.61** (1.17–5.81)	27	0.92 (0.63–1.34)
	85+	14	**2.20** (1.30–3.72)			2	**4.24** (1.06–17.0)	12	**2.00** (1.13–3.52)

Abbreviations: CI=confidence interval; SIR=standardised incidence ratio.

Results are presented for categories with observed cases. Only tumours diagnosed at least one year apart were included in the calculations.

Bold type represents a significant increase at the 5% confidence level.

**Table 4:** Summary of major risk factors that may result in the development of urinary bladder neoplasms in cancer patients

**Risk factor**	**Type of malignancy**
Smoking	Lung, larynx, oral cavity, oesophagus, anus, stomach, pancreas, cervix and kidney cancers
Workplace	Leukaemia, stomach and lung cancers (rubber workers)
exposures	Cancer in the nasal cavity (textile workers)
	Lung, renal pelvis and liver cancers (printing companies)
Chemotherapy	Lymphomas, leukaemia, ovary and breast cancers
Radiation therapy	Cervix, rectum, anus, testis and prostate cancers
Arsenic in water	Lung and liver cancers
